# Oxidative Stress and Changes of Important Metabolic Gene Expressions as a Potential Biomarker in the Diagnosis of Atherosclerosis in Leukocytes

**DOI:** 10.21470/1678-9741-2020-0378

**Published:** 2022

**Authors:** Mesut Işık, Abdullah Tunç, Şükrü Beydemir

**Affiliations:** 1 Department of Bioengineering, Faculty of Engineering, Bilecik Şeyh Edebali University, Bilecik, Turkey; 2 Department of Occupational Health and Safety, Faculty of Health Sciences, Bingöl University, Bingöl, Turkey; 3 Department of Biochemistry, Faculty of Pharmacy, Anadolu University, Eskişehir, Turkey

**Keywords:** Gene Expression, Coronary Artery Diseases, Biomarkers, Antioxidants, RNA, messenger, Atherosclerosis, Risk factors.

## Abstract

**Introduction:**

Presenilin 1 (PSEN1), catalase (CAT), glutathione-S-transferase (GST) and paraoxonase 1 (PON1) play a vital role in prediction, diagnosis and therapy of metabolic disorders.

**Methods:**

Metabolic enzyme activities and lipid peroxidation in serum of cerebrovascular diseases (CVD) and coronary artery diseases were measured by spectrophotometric methods. mRNA was isolated from leukocytes of the patient group and healthy adult patients. Quantitative gene expression of PSEN1, CAT and GST mRNA was identified by quantitative real-time polymerase chain reaction (qPCR).

**Results:**

The PSEN1, CAT and GST expression in patients showed significant differences compared to the control group. PSEN1 expression in leukocytes was significantly about twice as high as that of the control group in patients with CVD. The GST, CAT and PON1 activity showed significant differences in patient groups compared to the control group.

**Conclusion:**

The mRNA expression levels can be used as a potential biomarker in the diagnosis of atherosclerosis that occurs as a result of the metabolic disorder. In atherosclerotic patients, antioxidant status is independently related to an increased risk of cardiovascular events. Antioxidant activities and mRNA expressions may have predictive value, as well as available risk factors.

**Table:** 

Abbreviations, acronyms & symbols			
Aβ'	= Amyloid β fragment	MDA	= Malondialdehyde
CAD	= Coronary artery disease	PON1	= Paraoxonase 1
CAT	= Catalase	PSEN1	= Presenilin 1
CVD	= Cerebrovascular diseases	qPCR	= Quantitative real-time polymerase chain reaction
GPx	= Glutathione-S-peroxidase	ROS	= Reactive oxygen species
GST	=Glutathione-S-transferase	SOD	= Superoxide dismutase
HDL-C	= High-density lipoprotein cholesterol	SPSS	= Statistical Package for the Social Sciences
LDL-C	= Low-density lipoprotein cholesterol		

## INTRODUCTION

Oxidative stress is the unbalance between the manufacture and removal of reactive oxygen species (ROS), which are a family of molecules containing molecular oxygen and its derivatives produced in aerobic cells. ROS contain molecules such as hydroxyl radical, nitric oxide, hydrogen peroxide, superoxide anion and lipid radicals. The elimination of oxidative stress is accomplished by enzymatic defence mechanisms such as glutathione reductase, catalase (CAT), glutathione-S-peroxidase (GPx), glutathione-S-transferase (GST) and superoxide dismutase (SOD)^[1]^. Furthermore, there are ROS-scavenger thiol antioxidants such as reduced glutathione, glutaredoxin, thioredoxin and cysteine. These are low molecular mass antioxidants containing the (-SH) group and prevent the deformation of protein structures due to oxidative stress^[2]^. ROS induce progressive endothelial damage through alteration of extracellular matrix, apoptosis of endothelial cells, growth and migration of inflammatory cells and vascular smooth muscle^[3]^. The ROS were also revealed to be responsible for the increased expression of genes involved in immune and inflammatory responses. Recent studies have presented that oxidative stress conditions increase expression of genes encoding antioxidant enzyme activities such as g-glutamyl cysteine synthetase, heme oxygenase and GST^[4]^. Oxidative stress can suppress gene expression by changing a promoter sequence or by altering the activity of a transcription factor. Changes in RNA nucleotides due to oxidative stress have been reported to negatively affect protein expression^[5]^.

Many recent studies have shown that the oxidative stress induced by ROS in vascular and cardiac myocytes plays a key role in the development and pathogenesis of cardiovascular diseases such as atherosclerosis, cerebrovascular diseases (CVD), coronary artery disease (CAD), cardiac hypertrophy, hypercholesterolemia, ischemic heart disease, peripheral vascular disease, hypertension and heart failure^[6]^. Atherosclerosis mainly affects large and medium-sized elastic and muscular arteries and is the main systemic vascular disease triggering brain infarcts^[7]^.

Advanced age, male gender, obesity, hypertension, diabetes mellitus, elevated plasma lipoprotein and lipid levels, smoking, family history of cardiovascular disease (genetic transition) are well known for anticipating the risk of atherosclerosis^[7]^. Atherosclerosis and associated vascular diseases and risk factors such as physiological, environmental, and genetic factors can reason a wide-ranging simple and multifaceted vascular lesions in the brain^[8]^. Patients with cardiovascular disease generally present a number of vascular lesions such as microbleeds, microinfarcts and lipohyalinosis. Lipohyalinosis, the accumulation of hyaline in the connective tissue walls, hurts the entire vasculature. So, it affects the smaller vessels within the white matter^[9]^. These lesions are a direct result of atherosclerosis^[10]^. Moreover, these small vessel cerebral disorders are associated with the presenilin (PSEN) 1 gene that is responsible for the improvement of cerebral dementia such as Alzheimer's disease^[11]^. Individuals with genetic changes in one of the genes encoded for three transmembrane proteins (amyloid precursor protein, PSEN1, and PSEN2) deposit large amounts of the amyloid β fragment (Aβ) (1-42) in the brain and inevitably develop Alzheimer's disease. Aβ (1-42) induces DNA modification, lipid peroxidation, protein oxidation and ROS in synaptosomal or neuronal systems^[12]^. Thus, Aβ (1-42) might be seriously important in the oxidative stress sighted in Alzheimer's brain.

Oxidative stress is related to CVD and influences PON1 activities. The most important risk factor for atherosclerosis are blood lipids^[13]^ that contain low-density lipoprotein cholesterol (LDL-C), high-density lipoprotein cholesterol (HDL-C) triglyceride, free fatty acids, lipoproteins and total cholesterol. Actually, HDL-C concentration is also conversely associated with atherosclerosis. An important role of HDL-C functioning in the metabolism of lipid peroxides and their deposition on LDL-C is mediated by the PON1 enzyme that hydrolyses organophosphate substrates such as paraoxon^[14]^.

In this regard, the aim of this study is to determine the antioxidant metabolism and gene expression profiles in patients with CAD and CVD. Thus, the study specifically focuses on malondialdehyde (MDA) levels, CAT, GST and PON1 enzyme activities in plasma, and expression changes of various genes such as PSEN1, CAT and GST genes in leukocytes. We evaluated the relationship between oxidative stress caused by ROS and various gene expressions.

## METHODS

The study, conducted according to provisions of the Declaration of Helsinki, was approved by the Clinical Research Ethics Committee of the Erzurum Regional Training and Research Hospital. This study was conducted with groups, including 54 patients with CVD (27 females and 27 males, mean age 71.34), 60 patients with CAD (33 males and 27 females, mean age 72.34) and 69 healthy adults (33 females and 36 males, mean age 70.06). The patients with CVD in the study were acute ischemic stroke type. Those with a history of an inflammatory or infectious disease, autoimmune disorder, cancer, diabetes, smoking, haematological disorder, hepatic or renal disease or use of immunosuppressant, anti-inflammatory or anticoagulant drugs in the previous two months were excluded. Antiaggregant treatment (acetylsalicylic acid and/or clopidogrel) was applied to the patients. The antiaggregant agents, such as acetylsalicylic acid and clopidogrel, and anticoagulants, such as warfarin, are widely used in the primary and secondary prevention of cardio-cerebrovascular diseases and thromboembolic events.

In this study, serum samples previously obtained, and RNA isolated from leukocytes were used. Aliquots of this serum were kept frozen at -20 ºC until assayed. The activities of CAT, GST and MDA levels in healthy adults, control and patient groups were measured by spectrophotometric methods. The CAT, GST and PSEN1 mRNA quantitative gene expression in leukocytes was detected by real-time PCR.

### Sampling and RNA Extraction

Leukocytes from peripheral blood samples (2.5 mL in EDTA) were isolated by osmotic lysis method. Leukocyte RNA was extracted with the QIAamp RNA Blood Mini Kit provided by QIAGEN (Hilden, Germany), according to the manufacturer’s protocol. Each RNA sample was eluted with RNase-free water. The RNA that determined the concentration by measuring the absorbance at 280 nm was stored at -80 ºC.

### Reverse Transcription Polymerase Chain Reaction (RT-PCR)

The entire process of RT-PCR was performed according to the manufacturer’s procedure (Superscript III First-Strand Synthesis System for RT-PCR, Invitrogen). Thus, cDNA was synthesized.

### Real-Time PCR

Batch number of gene-specific primers and probes designed by QIAGEN were composed as JN137107 for GST, JN137109 for CAT, and JN137110 for PSEN1. As a template, 2 µL of the synthesized cDNA for real-time PCR was used. Multiplex real-time PCR was performed according to the manufacturer’s procedure (TaqMan FastStart Probe Master Mix, Roche). The experiment was performed in duplicates for both the GAPDH and the target gene as a housekeeping gene. Results of the target mRNA gene expression were expressed as 2^-∆∆Ct^, where ∆∆Ct=(Ct_AChE_-Ct_GAPDH_)p-(Ct_AChE_-Ct_GAPDH_)c. In this equation, c and p indicate the control and patient group, respectively.

### Measurement of GST Activity

GST activity was performed using a method modified by Harvey and Beutler with 1-chloro-2,4-dinitrobenzene (CDNB) as a substrate^[15]^. Reaction mixture containing 850 µL of 0.1 M phosphate buffer (pH 6.5), 50 µL of 20 mM GSH and 20 µL of 20 mM CDNB were pre-incubated 10 minutes at 20 ºC. The GST activity that started by adding 50 µL of serum was assayed spectrophotometrically, at 340 nm for 3 minutes by using a molar extinction coefficient of 9.6 mM/cm. The activity was expressed as units of activity (EU) per mg of protein^[15]^.

### Measurement of PON1 Activity

PON1 activity was determined at 25 ºC with paraoxon (diethyl p-nitrophenyl phosphate; 1 mM) in 50 mM glycine/NaOH (pH 10.5) containing 1 mMCaCl_2_. The paraoxonase enzyme assay was based on the estimation of p-nitrophenol at 412 nm. The molar extinction coefficient of p-nitrophenol (€=18.290 M^-1^cm^-1^ at pH 10.5) was used to calculate PON1 activity that catalyzes the hydrolysis of 1 mmol substrate at 25 ºC by using a spectrophotometer^[16]^.

### Catalase Activity

CAT activity performed by modifications of the Nelson and Kiesow method is based on the absorbance change at 240 nm for 2 minutes due to decomposition of H_2_O_2_^[17]^. The enzyme activity was calculated using the molar extinction coefficient (0.0436 cm^2^/µmol) and the results are expressed as enzyme units (EU).

### Determination of Lipid Peroxidation

The lipid peroxidation was estimated by the measurement of TBARS, as malondialdehyde (MDA, at 532 nm) and by modifications to the method of Jentzsch et al.^[18]^. The results are expressed as µmol MDA/L of serum.

### Statistical Analysis

Experiments were performed twice for each assay. Statistical analysis was done by the Statistical Package for the Social Sciences software version 13.0 (SPSS Inc., Chicago, IL, USA). Summary statistics of the study groups were presented as mean ± standard deviation. Data were analysed statistically by one-way ANOVA followed by LSD (least significant difference) multiple tests. Differences were considered significant when *P*<0.05.

## RESULTS

**Fig. 1:**
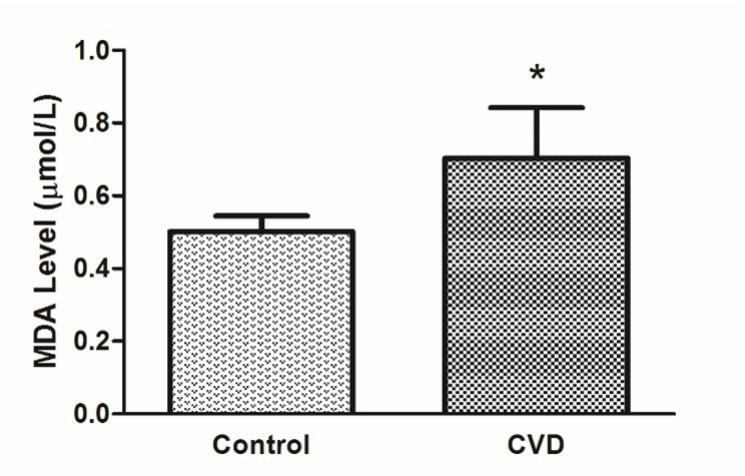
Determination of malondialdehyde level as a biomarker of oxidative stress in plasma for all groups (controls and patients with CVD). The symbols denote significant differences from the control group at (*) P<0.05 by using one-way ANOVA with LSD (least significant difference) post hoc test.

MDA levels in blood plasma from patients with CVD were investigated. In this context, the MDA level in the CVD group was significantly higher (*P*<0.05) than in the control group (Figure 1).

**Fig. 2:**
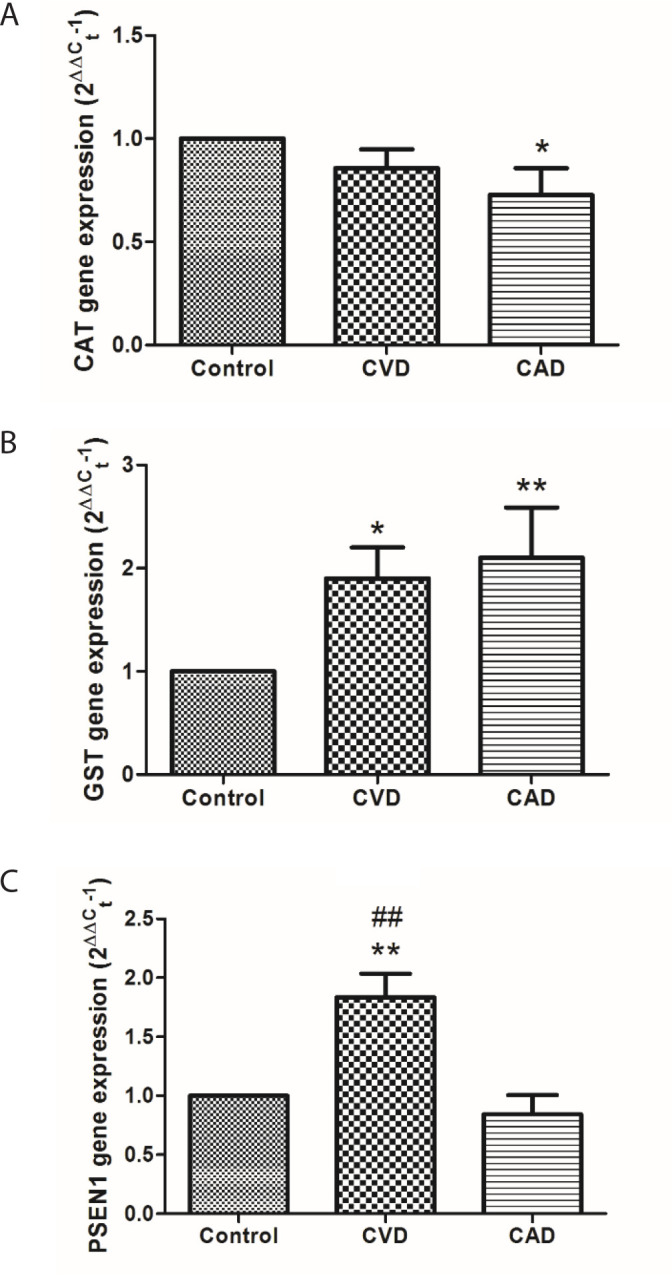
CAT (a), GST (b) and PSEN1 (c) gene expression levels in leukocyte for all groups (patients with CVD, CAD and controls). The symbols denote significant differences from the control group at (*) P<0.05 and (**) P<0.01 from the CAD group at (##)P<0.01 by using one-way ANOVA with LSD (least significant difference) post hoc test.

CAT expression level in leukocyte samples taken from the CAD group was significantly lower (*P*<0.05) than in the control group (Figure 2a). When compared to control, the GST expression level in the CVD (*P*<0.05) and CAD groups (*P*<0.01) was significantly higher (almost 2 times) in leukocytes (Figure 2b). The expression of PSEN1 in leukocytes was significantly about twice as high as that of the control group and CAD group in patients with CVD (*P*<0.01) (Figure 2c).

**Fig. 3:**
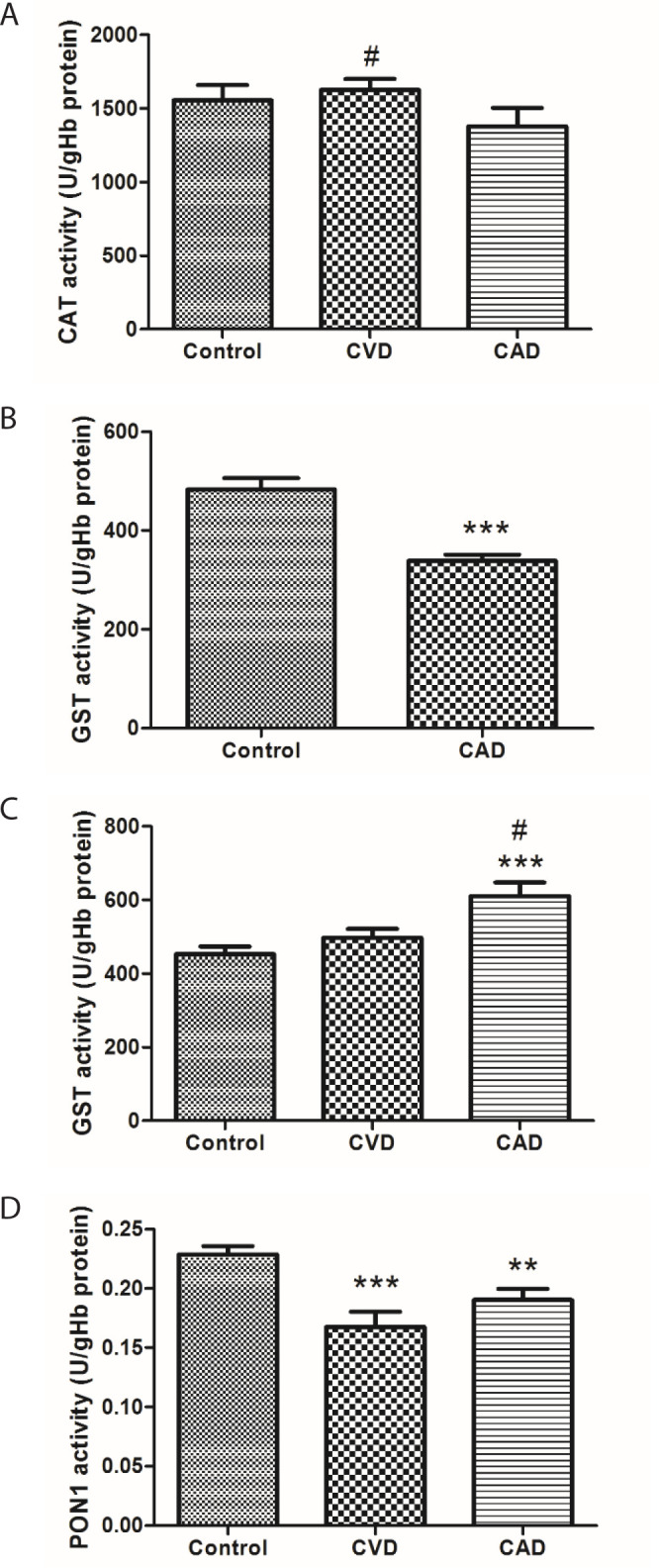
CAT (a), GST (b) and PSEN (d) enzyme activity levels in plasma and GST (c) enzyme activity in erythrocytes for all groups (patients with CVD and CAD and controls). The symbols denote significant differences from the control group at (**) P<0.01 and (***) P<0.001 from the CAD group at (#) P<0.05 by using one-way ANOVA with LSD (least significant difference) post hoc test

Plasma CAT activity in CVD and CAD groups was not significantly different from the control group (*P*>0.05), whereas activity in the CVD group was significantly higher (*P*<0.05) than in the CAD group (Figure 3a). The plasma GST activity level in the CAD group (*P*<0.001) was significantly lower according to the control group (Figure 3b). GST activity in erythrocytes of patients with CAD was higher than in the control group (*P*<0.001) and in the CVD group (*P*<0.05) (Figure 3c). Plasma PON1 activity in the control group was higher than in patients with CVD (*P*<0.001) and CAD (*P*>0.01) (Figure 3d).

## DISCUSSION

Aerobic organisms continuously produce ROS in their normal metabolic processes. Antioxidant metabolism provides cellular redox balance. If the balance between these two cases conditions is deteriorated, the prooxidant state that occurs is called oxidative stress. ROS execute by attacking carbohydrates, lipids and nucleic acids^[19]^.

This study and our other studies showed that the baseline levels and the degrees of induction observed in CVD patients differed among the oxidative stress markers. Interestingly, there was a correlation between the baseline levels of these oxidative stress biomarkers. MDA levels were linked with the index of atherosclerotic diseases. These findings promote that this oxidative stress marker reflects the different characteristics of oxidative stress. Several studies have confirmed increased levels of MDA in the serum of acute atherosclerotic patients^[20^,^21]^.

Malondialdehyde (MDA) is a product of the peroxidation of low-density lipoprotein (LDL) fatty acids. It is considered as the biomarker of lipid peroxidation. Increased levels of MDA in blood tissues in patients with CVD indicate oxidative stress damage^[20]^. In our study, the increase in MDA level in CVD patients suggests the presence of oxidative stress. In our previous studies, we investigated PCO and T-SH levels in CAD and CVD patients and found that T-SH levels were lower and PCO levels were higher in these patients compared to control groups^[21]^. These conditions are an indicator of oxidative damage in CVD and CAD patients. An increase in MDA levels may indicate that it is the result of damage to membrane lipids. Our study indicates that the lipid metabolism processes in CAD and CVD patients are damaged.

GST activity in CAD patients in our present study and in CVD patients in our previous study resulted in a significant reduction^[21]^. Also, Dubois et al. proved that there was a significant reduction in patients with unstable angina and chronic heart failure. Several alterations in GST activity and GSH metabolism can affect various signalling events that have caused the development of different diseases^[22]^. Hence, GST has been expected to play an important role in various diseases.

In our study, CAT activity (although not significant) decreased in patients with CAD. This is a visible and important phenomenon in the pathogenicity of heart diseases. Enzyme activity levels in patients with CAD may be the evidence of increased oxidative capacity, which in a study found that CAT activity was significantly lower in staged patients than in control patients^[23]^. In the *ex vivo* study in rat hearts, cardiac dysfunction and ischemia-reperfusion were also performed. It was found that ischemia-reperfusion injury improved when treated with CAT^[6]^.

Low expression and activity of CAT in CAD means that H_2_O_2_ levels are low. PON enzyme activity is therefore expected to be low. PON1 is known to have an atherogenic protective role. This role is fulfilled by its cytoprotective role, preventing LDL lipid peroxidation. Serum expression of PON1 is downregulated by oxidative stress^[24^,^25]^. A study showed that paraoxonases decrease oxidative stress in tissues and serum and defend against cardiovascular diseases^[26]^. Therefore, a decrease in PON1 activity is also expected in this process. The decrease in the activity of this enzyme in both CVD and CAD patients was caused by the presence of oxidative stress. Accordingly, our study shows that PON1 provides atherosclerotic protection in plasma.

**Fig. 4:**
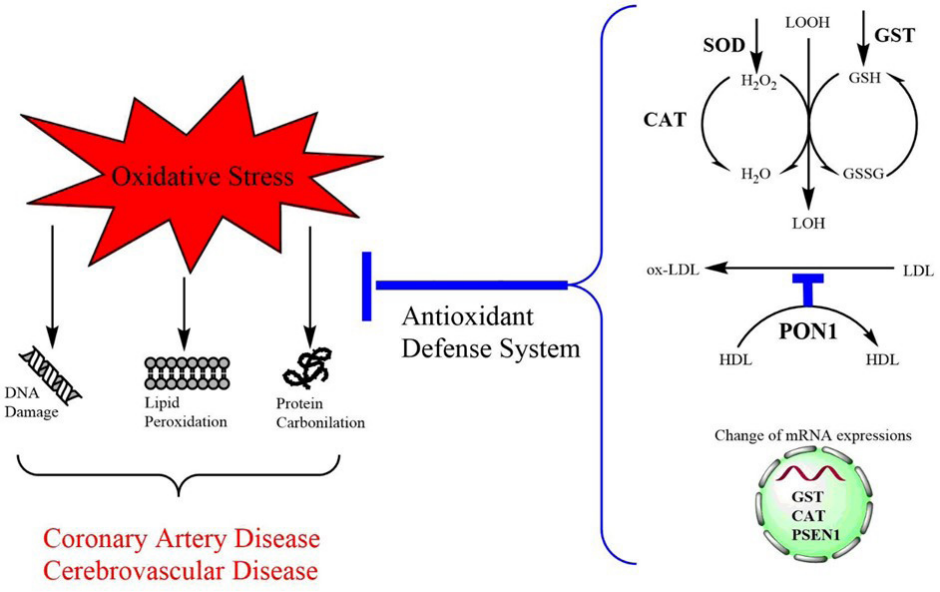
Atherosclerosis formation due to DNA, lipid and protein oxidation, the role of antioxidant defence system and gene expression mechanisms in decrease of oxidative stress.

Analysis of mRNA levels applied by real-time PCR is more delicate and isoform-specific than enzyme activity analysis. Though, whereas decreased mRNA levels are probable to be attended by a decrease in protein expression, an increase in mRNA levels does not constantly denote increased protein expression and enzyme activities^[27]^. In our study, CAT expression levels were significantly lower in CAD patients compared to controls but did not decrease significantly in patients with CVD. This is partly similar to enzyme activity. Increased leukocyte GST mRNA levels reciprocated with a decrease in plasma and an increase in erythrocytes in enzyme activity. In this context, an increase in CAT and GST mRNA levels were found to be correlated with enzyme activity levels. This means that the body endeavours to tolerate cardiac damage. At least this conclusion may come to mind. This may result in a decrease in the expression of antioxidant enzyme genes in the case of atherosclerosis (Figure 4). Hoen et al. also observed that antioxidant enzyme gene expressions such as CAT were significantly reduced during atherosclerosis in ApoE-deleted mice^[28]^. It may be thought that increased levels of GST mRNA in erythrocytes in CAD is induced by the fight against increased oxidative stress, thereby creating a protection mechanism. The ROS production that plays a role in transactivation or suppression of a gene promoter can also specifically downregulate the expression of various genes^[5]^. A study has reported that while an increase in GPx and Cu-Zn SOD mRNA expression parallels an increase in the activity of enzymes, cells may be prevented from oxidative stress with excessive gene expression as opposed to a decrease in enzyme activity^[29]^.

Oxidative stress promotes the formation of neurofibrillary tangles. In fact, it has been proven by experiments that oxidative stress is a predictable phenomenon in the pathogenesis of AD. Amyloid β proteins, which are caused by abnormalities in the *PSEN1* gene, which play an active role in AD, are also thought to play a critical role in oxidative stress^[30]^. Shoeb et al. showed increased expression of the *PSEN1* gene by oxidative stress^[31]^. In our study, *PSEN1* gene expression was significantly increased in CVD patients compared to control patients, which may be directly related to oxidative stress. It was researched for the first time that PSEN had also increased in CVD patients. Thus, there is a close relationship between cerebrovascular atherosclerosis and neurodegenerative dementia.

## CONCLUSION

We evaluated the relationship between quantitative gene expressions and activities of pro- and antioxidant enzymes in patients with CAD and cardiovascular diseases. These findings suggest that CAD and CVD status may be determined by metabolic oxidative stress. *PSEN1* gene expression changes can also be considered as an important metabolic marker in CAD and CVD. In other words, the fact that oxidative stress plays a major role in CAD and CVD has been confirmed by our results. The cells can disregard moderate oxidative damage by altering enzyme activity and gene expression (Figure 4).

**Table:** 

Authors' roles & responsibilities	
MI	Substantial contributions to the conception or design of the work; or the acquisition, analysis, or interpretation of data for the work; final approval of the version to be published
AT	Substantial contributions to the conception or design of the work; or the acquisition, analysis, or interpretation of data for the work; final approval of the version to be published
ŞB	Substantial contributions to the conception or design of the work; or the acquisition, analysis, or interpretation of data for the work; final approval of the version to be published
